# Genomic Diversity of the *tet*(X)-Positive *Myroides* Species

**DOI:** 10.3390/microorganisms13061180

**Published:** 2025-05-22

**Authors:** Chong Chen, Taotao Wu, Jing Liu, Yilin Lv

**Affiliations:** 1 Joint International Research Laboratory of Agriculture and Agri-Product Safety, Institutes of Agricultural Science and Technology Development, Yangzhou University, Yangzhou 225009, China; wtt1493270230@163.com (T.W.); 17851971707@163.com (J.L.); 15050731151@163.com (Y.L.); 2 Jiangsu Key Laboratory of Zoonosis, Jiangsu Co-Innovation Center for Prevention and Control of Important Animal Infectious Diseases and Zoonoses, Yangzhou University, Yangzhou 225009, China; 3 Key Laboratory of Prevention and Control of Biological Hazard Factors (Animal Origin) for Agrifood Safety and Quality, Yangzhou University, Yangzhou 225009, China

**Keywords:** *Myroides* spp., tetracyclines, *tet*(X), macrolides, *estT*, IS*CR2*

## Abstract

The rapid spread of *tet*(X) genes capable of inactivating tigecycline represents a critical challenge to global public health. This study aims to explore the distribution, genetic diversity, and transferability of *tet*(X) genes in *Myroides*, a genus of Gram-negative bacteria increasingly implicated in multidrug-resistant (MDR) bacterial infections. From 2021 to 2024, 646 samples of chicken, sheep, soil, and water were randomly collected, yielding nine chicken-derived *tet*(X)-positive *Myroides* sp. strains in Shandong, China. All of them were MDR to tetracycline, ceftazidime, gentamicin, amikacin, colistin, ciprofloxacin, gatifloxacin, and trimethoprim-sulfamethoxazole, with elevated minimum inhibitory concentrations (MICs) for tigecycline, florfenicol, and macrolides, but exhibited susceptibility to meropenem (100%), ampicillin-sulbactam (66.7%), and cefotaxime (33.3%). A genomic analysis of the isolates and 86 public *tet*(X)-positive *Myroides* genomes revealed the widespread distribution of *tet*(X) and macrolide-inactivating *estT* genes across 12 *Myroides* species, including 7 novel species. Eight *tet*(X) and eight estT variants were identified, half of which were novel. The phylogenetic analysis highlighted interspecies transmission risks, with IS*CR2*-mediated transposons of *tet*(X6) and *estT-2* across *Myroides*, *Riemerella*, *Empedobacter*, *Providencia*, *Acinetobacter*, and *Proteus* species. These findings illuminate the genomic diversity driving antibiotic resistance in understudied bacterial taxa, with implications for global One Health strategies.

## 1. Introduction

*Myroides*, a genus of non-motile, non-fermenting, aerobic, and Gram-negative bacteria belonging to *Flavobacteriaceae*, comprises 17 validly published and correct species [1,2]. Traditionally, these bacteria are considered non-pathogenic, but recent clinical reports have revealed their pathogenicity, particularly in immunocompromised patients [2,3,4,5]. While the infections caused by *Myroides* spp. of clinical significance remain limited in scope, surveillance studies show increasing detection rates that may be attributable to advances in molecular diagnostic techniques [3]. Notably, clinically relevant *Myroides* species such as *Myroides odoratimimus* and *Myroides odoratus* demonstrate extensive antimicrobial resistance profiles encompassing β-lactam and aminoglycoside antibiotics, thereby exacerbating therapeutic limitations and emerging as a growing concern in nosocomial infection [2,6,7,8].

As a third-generation tetracycline derivative and the first glycylcycline antibacterial agent, tigecycline holds critical status as a last-line therapeutic option against MDR bacterial pathogens in clinical settings [9]. The compound received initial Food and Drug Administration approval in 2005 for management of complicated intra-abdominal infections and acute bacterial skin/skin structure infections, followed by expanded indications for community-acquired bacterial pneumonia in 2009 [10]. Subsequent regulatory approvals were obtained in the European Union (2006) and China (2011), establishing its global therapeutic application framework supported by accumulating clinical evidence [11,12]. Since the first report in 2019, a variety of tigecycline resistance genes *tet*(X) have been reported in *Enterobacteriaceae*, *Acinetobacter* spp., *Empedobacter* spp., *Elizabethkingia* spp., *Riemerella anatipestifer*, and *Pseudomonas caeni* in China [13,14,15,16,17,18,19,20,21].

Recently, the *tet*(X) genes were reported in *M. odoratimimus*, *M. odoratus*, and *Myroides phaeus* from inpatient, pig, fish, and soil samples [6,8,13,22,23]. However, the genetic diversity and transferability of *tet*(X) genes in *Myroides* spp. remained poorly understood. In this study, we intend to explore the prevalence, antimicrobial susceptibility, phylogenetic relationship, and transmission risk of *tet*(X)-positive *Myroides* sp. isolates, together with blast querying in the public database.

## 2. Materials and Methods

### 2.1. Sample Collection and Bacterial Isolation

During a surveillance across three Chinese provinces, Shandong, Jiangsu, and Ningxia, 646 non-redundant samples were randomly collected from 2021 to 2024, comprising chicken feces (n = 449), sheep feces (n = 95), and surrounding environmental samples (soil, n = 60; water, n = 42). Specimens were homogenized in 0.9% sterile saline (1:5 *w*/*v* for solids; 1:5 *v*/*v* for liquids) with vortex mixing, and 100 μL of supernatant was plated on Luria–Bertani (LB) agar plates supplemented with tigecycline (4 mg/L) for selective isolation of tigecycline-resistant strains. Presumptive *tet*(X)-positive *Myroides* sp. strains underwent molecular confirmation via PCR amplification of conserved 16S rRNA regions and *tet*(X)-specific primers, followed by bidirectional Sanger sequencing [24,25].

### 2.2. Antibiotic Resistance Evaluation

MICs were determined by two-fold Mueller–Hinton (MH) agar dilution according to the Clinical and Laboratory Standards Institute guidelines [26]. Both *Myroides* spp. and *Acinetobacter* spp. are non-motile, non-fermenting, aerobic, and Gram-negative bacteria [2,27], and therefore, *Acinetobacter* spp. breakpoints were used for interpreting MICs of *Myroides* spp. in this study, which lacked the standardized breakpoints. These antibiotics (Yuanye, Shanghai, China) included tetracycline (dilution range, 0.5–256 mg/L), amikacin (0.5–256 mg/L), gentamicin (0.25–256 mg/L), ciprofloxacin (0.0039–64 mg/L), gatifloxacin (0.0078–16 mg/L), colistin (0.25–256 mg/L), trimethoprim-sulfamethoxazole (0.5/9.5–16/304 mg/L), ampicillin-sulbactam (0.125/0.0625–256/128 mg/L), ceftazidime (0.125–256 mg/L), cefotaxime (0.03125–256 mg/L), meropenem (0.0078–64 mg/L), tigecycline (0.03125–64 mg/L), florfenicol (2–256 mg/L mg/L), tylosin (0.25–512 mg/L), tilmicosin (0.125–256 mg/L), and tildipirosin (0.125–256 mg/L), of which the last five antibiotics lacked resistance breakpoints. For quality standardization, the reference strain *Escherichia coli* ATCC 25922 was systematically incorporated as the antimicrobial susceptibility testing control organism.

### 2.3. Whole Genome Sequencing (WGS)

The genomic landscape of *tet*(X)-harboring *Myroides* sp. isolates was characterized through integrated sequencing strategies. Primary sequencing was conducted using the Illumina NovaSeq 6000 platform (ANOROAD, Beijing, China) with 2 × 150 bp paired-end sequencing, followed by de novo assembly via SPAdes version 3.15.5 (Russian Academy of Sciences, St. Petersburg, Russia) [28]. *M. odoratimimus* C26-4 and C34-1 then underwent third-generation sequencing using Oxford Nanopore PromethION (BENAGEN, Wuhan, China) with ultra-long read strategies, achieving circularized genomes through hybrid assembly with Unicycler version 0.5.1 (University of Melbourne, Melbourne, Australia) [29]. Complementary to experimental data, all publicly available WGS data of *tet*(X)-positive *Myroides* sp. strains were retrieved from the NCBI database [30]. All the genome assemblies were checked by CheckM version 1.1.6 (University of Queensland, Brisbane, Australia) and Quast version 5.2.0 (Russian Academy of Sciences, St. Petersburg, Russia), which were defined by <350 contigs, >50 kb N50, >95% genome completeness, <2 genome contamination, and <50 genome heterogeneity [31,32].

### 2.4. Bioinformatics Analyses

Genomic analyses were conducted using IPGA version 1.09 (Chinese Academy of Sciences, Beijing, China) to determine average nucleotide identity (ANI) and construct a core single-nucleotide polymorphism (SNP) phylogeny for *tet*(X)-positive *Myroides* spp., with species delineation based on a >95% ANI threshold against LPSN type strains [33,34,35]. All *Myroides* genomes were further confirmed by in silico DNA–DNA hybridization (*is*DDH) analyses and classified by a >70% *is*DDH threshold [35]. Antibiotic resistance genes (ARGs) and virulence factors were identified by >80% identity and >60% coverage thresholds via ABRicate version 1.0.1 (University of Melbourne, Melbourne, Australia), with phylogeny-heatmap integration performed using ggtreeExtra (Southern Medical University, Guangzhou, China) [36,37]. Maximum likelihood trees of *tet*(X) or *estT* variants were generated by MEGA-X version 10.1.8 (Pennsylvania State University, State College, PA, USA) in 500 bootstrap replicates and visualized by FigTree version 1.4.4 (University of Edinburgh, Edinburgh, UK), with novel alleles defined by ≥2% amino acid divergence according to international standards for resistance gene naming [18,38,39]. Three-dimensional structural prediction of Tet(X) and EstT variants was performed through SWISS-MODEL using the experimentally resolved Tet(X2)-tigecycline co-crystal structure (PDB accession number: 4A6N) and our previously simulated structure of EstT-1, respectively [18,40]. Genome annotation utilized RAST version 2.0 (Fellowship for Interpretation of Genomes, Burr Ridge, IL, USA), while chromosomal comparisons of *tet*(X)-bearing regions employed BRIG version 0.95 (University of Queensland, Brisbane, Australia) [41,42]. Genetic contexts of *tet*(X) or *estT* genes were achieved through Easyfig version 2.2.5 (University of Queensland, Brisbane, Australia) [43].

### 2.5. Phenotypic Experiments of Novel Myroides Species

Morphology of the novel *Myroides* species we isolated was determined on LB agar plates at 35 °C for 24 h, and through Gram-staining [44]. Anaerobic growth was examined on LB agar plates in an anaerobic bag at 35 °C for 24 h. Growth at different NaCl concentrations (0%, 1%, 2%, 3%, 4%, 5%) or pH (4, 5, 6, 7, 8, 9, 10, 11, 12) was performed in LB broth at 35 °C for 24 h. Growth at various temperatures (20 °C, 25 °C, 30 °C, 35 °C, 37 °C, 38 °C, 39 °C, 40 °C, 41 °C, 42 °C) was tested on LB agar plates for 24 h. Bacterial motility was tested in LB medium with 0.4% agar at 35 °C for 24 h. Hemolysis was examined on LB agar plates containing 5% sheep blood at 35 °C for 24 h. Physiological activities on glucose, oxidase, citrate, maltose, arginine dihydrolase, mannitol, xylose, nitrate reduction, DNA, and acetamide were detected by a non-fermenting bacterial identification kit (HuanKai, Guangzhou, China). *M. odoratimimus* ATCC BAA-634 was used as the reference standard.

### 2.6. Cloning Expression

The novel *tet*(X) and *estT* variants we identified were directionally cloned into the L-arabinose-inducible pBAD24 expression vector with *EcoR* I/*Sal* I and *Nhe* I/*Sal* I restriction sites (Table S1), respectively, followed by electroporation into chemically competent *E. coli* JM109 cells. Transformants were selected on LB agar under ampicillin pressure (100 mg/L) and validated through colony PCR and bidirectional Sanger sequencing [45]. For MIC determination, log-phase cultures induced with 0.1% L-arabinose were subjected to two-fold broth microdilution, with tetracyclines (tetracycline/doxycycline/minocycline/tigecycline) or macrolides (tylosin/tilmicosin/tildipirosin) tested [26]. Isogenic control strains harboring *tet*(X2), *tet*(X6), *tet*(X6)-*tet*(X2), *estT-1.2*, *estT-2*, or empty vector were included for phenotypic benchmarking [18,20].

### 2.7. Transfer of tet(X) Genes

As documented in our prior research [18], the horizontal transfer potential of *tet*(X)-mediated tigecycline resistance was evaluated through biparental conjugation assays using rifampicin-resistant *Acinetobacter baylyi* ADP1 and *E. coli* C600 as recipients. Following 16-h incubation at a donor-to-recipient ratio of 1:3, putative transconjugant colonies were isolated on selective LB agar plates supplemented with tigecycline (2 mg/L) and rifampin (125 mg/L). All candidate colonies subsequently underwent molecular confirmation through *tet*(X)-specific PCR amplification, followed by species-specific PCR fingerprinting for *A. baylyi* identification and enterobacterial repetitive intergenic consensus PCR (ERIC-PCR) genotyping for *E. coli* validation [46,47]. In addition, natural transformation was conducted to explore the transferability of *tet*(X) into *A. baylyi* ADP1, *E. coli* C600, and *M. odoratimimus* ATCC BAA-634 [48].

## 3. Results

### 3.1. Sporadic Detection of tet(X)-Positive MDR Myroides spp.

**Figure 1:**
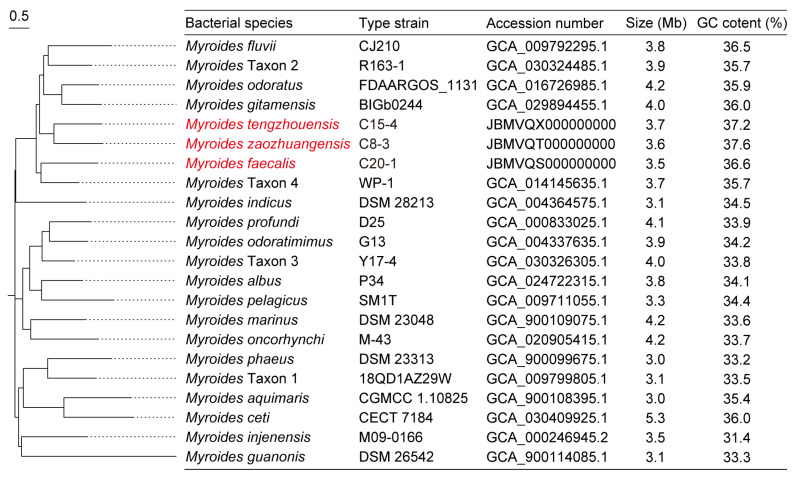
SNP-based phylogenetic tree of 22 different *Myroides* species. There are seven novel species named *Myroides tengzhouensis*, *Myroides zaozhuangensis*, *Myroides faecalis*, and *Myroides* Taxon 1–Taxon 4, respectively, of which the novel *Myroides* species we isolated are marked in red. Type strains and their GenBank accession numbers, genome sizes, and GC contents are present in parallel. Bar, 0.5 nucleotide substitutions per site.

In this study, nine *tet*(X)-positive *Myroides* sp. strains were isolated from chicken samples in Shandong, but were negative in sheep, soil, and water samples in Shandong, Jiangsu, and Ningxia, China. These isolates contained five *M. odoratimimus* and four novel *Myroides* sp. strains of *Myroides tengzhouensis* (n = 1), *Myroides faecalis* (n = 2), and *Myroides zaozhuangensis* (n = 1), which shared less than 95% intra-species ANI threshold and 70% intra-species *is*DDH threshold with the reference *Myroides* species (Figure 1). As shown in Table 1, MICs of 16 antibiotics were tested, of which 12 antibiotics (except florfenicol, tylosin, tilmicosin, and tildipirosin) have been approved for the treatment of human infections in China. All of these strains were MDR to tetracycline, ceftazidime, gentamicin, amikacin, colistin, ciprofloxacin, gatifloxacin, and trimethoprim-sulfamethoxazole but exhibited susceptibility to meropenem (100%), ampicillin-sulbactam (66.7%), and cefotaxime (33.3%). Meanwhile, MIC_90_ of tigecycline, florfenicol, tylosin, tilmicosin, and tildipirosin were 16 mg/L, 64 mg/L, 512 mg/L, 64 mg/L, and 32 mg/L, respectively.

**Figure 2:**
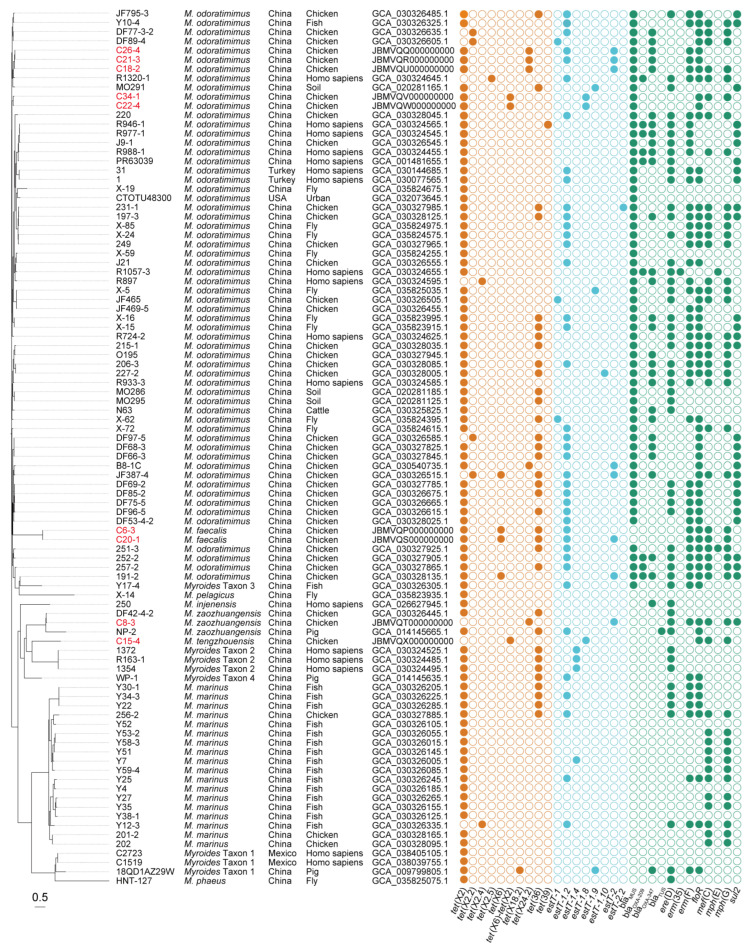
Phylogeny and ARGs of *tet*(X)-positive *Myroides* sp. strains. Bacterial species, geographical location, sampling sources, and GenBank accession numbers are present in parallel. The *tet*(X)-positive *Myroides* sp. strains we isolated are marked in red. In addition, the *tet*(X) and *estT* genes are colored in brown and cyan, respectively, and the remainder are colored in green. Bar, 0.5 nucleotide substitutions per site.

By querying the public NCBI database (Figure 2), another 86 *tet*(X)-positive *Myroides* sp. strains were collected in China (n = 81), Turkey (n = 2), Mexico (n = 2), and the USA (n = 1). In brief, the *tet*(X) genes were widely distributed in 10 different *Myroides* species, such as *M. odoratimimus* (n = 55), *Myroides marinus* (n = 18), *M. phaeus* (n = 1), *Myroides pelagicus* (n = 1), *Myroides injenensis* (n = 1), and five novel *Myroides* species of *M. zaozhuangensis* (n = 2), *Myroides* Taxon 1 (n = 3), *Myroides* Taxon 2 (n = 3), *Myroides* Taxon 3 (n = 1), and *Myroides* Taxon 4 (n = 1). Source tracing of bacterial strains indicated chicken (n = 33) is the main reservoir of *tet*(X)-positive *Myroides* spp., followed by homo sapiens (n = 17), fish (n = 17), fly (n = 11), pig (n = 3), soil (n = 3), cattle (n = 1), and urban (n = 1) samples. Despite the lack of sample sizes and MIC data, these online genomes indicated the sporadic detection and bacterial diversity of *Myroides* sp. strains carrying *tet*(X) genes worldwide.

### 3.2. Polymorphism of tet(X) and estT

Computational resistance gene profiling of 95 *tet*(X)-harboring *Myroides* sp. strains revealed five classes of ARGs for tetracyclines, β-lactams, macrolides, phenicols, and sulfonamides, of which 11.6% carried two *tet*(X) variants (Figure 2). Among eight *tet*(X) variants characterized, four were previously annotated ones encompassing *tet*(X2) (n = 86), *tet*(X2.2) (n = 4), *tet*(X6) (n = 4), and *tet*(X6)-*tet*(X2) (n = 3). The remaining four novel variants were designated under standardized nomenclature as *tet*(X2.4) (n = 2), *tet*(X2.5) (n = 1), *tet*(X18.2) (n = 1), and *tet*(X24.2) (n = 5). Particularly, *tet*(X2) (n = 7), *tet*(X6) (n = 2), *tet*(X6)-*tet*(X2) (n = 3), and *tet*(X24.2) (n = 4) were identified in our epidemiological investigation. Functional validation of them through heterologous expression in *E. coli* JM109 demonstrated 2–64-fold MICs against tetracycline, doxycycline, minocycline, and tigecycline compared to an isogenic empty-vector control (Table 2). Homology modeling confirmed Tet(X6), Tet(X6)-Tet(X2), Tet(X18.2), and Tet(X24.2) with a lack of 10 N-terminal amino acids shared six key amino acid residues at S272, M319, T329, N330, I340, and E341, as previously reported [49,50], leading to a high-level resistance phenotype for tetracyclines, while *tet*(X2), *tet*(X2.2), *tet*(X2.4), and *tet*(X2.5) exhibited a low-level activity (Figure S1).

It is noted that 58 (61.1%) out of 95 *tet*(X)-positive *Myroides* sp. strains carried *estT* genes, of which 10.3% carried two *estT* variants (Figure 2). These variants included *estT-1* (n = 3), *estT-1.2* (n = 40), *estT-1.4* (n = 4), *estT-2* (n = 9), and four novel variants (n = 8). According to the gene assignment rule, the novel variants were designated as *estT-1.8* (n = 3), *estT-1.9* (n = 3), *estT-1.10* (n = 1), and *estT-2.2* (n = 1). Particularly, *estT-1.2* (n = 4), *estT-1.8* (n = 3), and *estT-2* (n = 6) were identified in our epidemiological investigation. MIC results showed *E. coli* clones of them that exhibited 2–4-fold increases for 16–atom–containing tylosin, tilmicosin, and tildipirosin of macrolides compared to an isogenic empty-vector control, suggesting a low-level activity (Table 2). Homology modeling of eight EstT proteins was also conducted, with a lack of N-terminal random coil of EstT-1, and it remains to be studied (Figure S2).

### 3.3. Novel Myroides Species

To date, a total of 22 *Myroides* species have been reported with different genome sizes but have a similar GC content ranging from 31.4% to 37.6% (Figure 1). The physiological characteristics of novel species *M. zaozhuangensis* C8-3, *M. tengzhouensis* C15-4, and *M. faecalis* C20-1 were analyzed against *M. odoratimimus* ATCC BAA-634 and described as below (Table S2).

#### 3.3.1. Description of *Myroides zaozhuangensis* sp. nov.

*Myroides zaozhuangensis* (zao.zhuang.en’sis. N.L. masc. adj. *zaozhuangensis*, referring to Zaozhuang, Shandong, China).

Cells are Gram-staining-negative, rod-shaped, aerobic, non-motile, and non-hemolytic. Colonies are light yellow and circular, with a smooth surface and regular margin after 24 h of incubation at LB agar. It survives at pH 5–9, NaCl up to 4%, and temperature up to 41 °C. The tests are negative for glucose, citrate, maltose, mannitol, xylose, and nitrate reduction, but positive for oxidase, arginine dihydrolase, DNA hydrolysis, and acetamide utilization.

The type strain C8-3^T^ (GDMCC 66301) was isolated from a chicken manure sample in 2021 in Shandong, China. The size of whole-genome sequences of the type strain is 3.6 Mb, with a G + C content of 37.6%, which has been deposited in the NCBI database under the GenBank accession number: JBMVQT000000000.

#### 3.3.2. Description of *Myroides tengzhouensis* sp. nov.

*Myroides tengzhouensis* (teng.zhou.en’sis. N.L. masc. adj. *tengzhouensis*, referring to Tengzhou, Shandong, China).

Cells are Gram-staining-negative, rod-shaped, aerobic, non-motile, and non-hemolytic. Colonies are light yellow and circular, with a smooth surface and regular margin after 24 h of incubation on LB agar. It survives at pH 6–8, NaCl up to 3%, and temperature up to 38 °C. The tests are negative for glucose, citrate, maltose, mannitol, xylose, and nitrate reduction, but positive for oxidase, arginine dihydrolase, DNA hydrolysis, and acetamide utilization.

The type strain C15-4^T^ (GDMCC 66292) was isolated from a chicken manure sample in 2021 in Shandong, China. The size of whole-genome sequences of the type strain is 3.7 Mb, with a G + C content of 37.2%, which has been deposited in the NCBI database under the GenBank accession number: JBMVQX000000000.

#### 3.3.3. Description of *Myroides faecalis* sp. nov.

*Myroides faecalis* (fae.ca’lis. N.L. masc. adj. *faecalis*, pertaining to feces).

Cells are Gram-staining-negative, rod-shaped, aerobic, non-motile, and non-hemolytic. Colonies are light yellow and circular, with a smooth surface and regular margin after 24 h of incubation on LB agar. It survives at pH 6–8, NaCl up to 4%, and temperature up to 38 °C. The tests are negative for glucose, citrate, maltose, mannitol, xylose, and nitrate reduction, but positive for oxidase, arginine dihydrolase, DNA hydrolysis, and acetamide utilization.

The type strain C20-1^T^ (GDMCC 66293) was isolated from a chicken manure sample in 2021 in Shandong, China. The size of whole-genome sequences of the type strain is 3.5 Mb, with a G + C content of 36.6%, which has been deposited in the NCBI database under the GenBank accession number: JBMVQS000000000.

### 3.4. Phylogeny of Myroides spp.

Following SNP analyses, an SNP-based phylogenetic tree of 95 *tet*(X)-positive *Myroides* sp. strains was conducted for bacterial relationship (Figure 2). The tree revealed *M. marinus*, *M. phaeus*, *M. pelagicus*, *M. injenensis*, *M. tengzhouensis*, *M. zaozhuangensis*, and *Myroides* Taxon 1–Taxon 4 formed 10 separate clusters, except the complex of *M. odoratimimus* and *M. faecalis*. All the novel *Myroides* species shared 179-4099 SNPs with the reference strain *M. odoratimimus* G13 (GenBank accession number: GCA_004337635.1), and there existed a novel bacterial evolutionary branch consisting of *M. tengzhouensis*, *M. zaozhuangensis*, *Myroides* Taxon 2, and *Myroides* Taxon 4. Scarcely, the clonal transmission risk of *tet*(X) genes occurred in *M. odoratimimus*, *M. marinus*, *M. faecalis*, *M. zaozhuangensis*, *Myroides* Taxon 1, or *Myroides* Taxon 2, indicating the potential importance of the horizontal transmission route.

### 3.5. ISCR2-Mediated Transposons of tet(X) and estT

**Figure 3:**
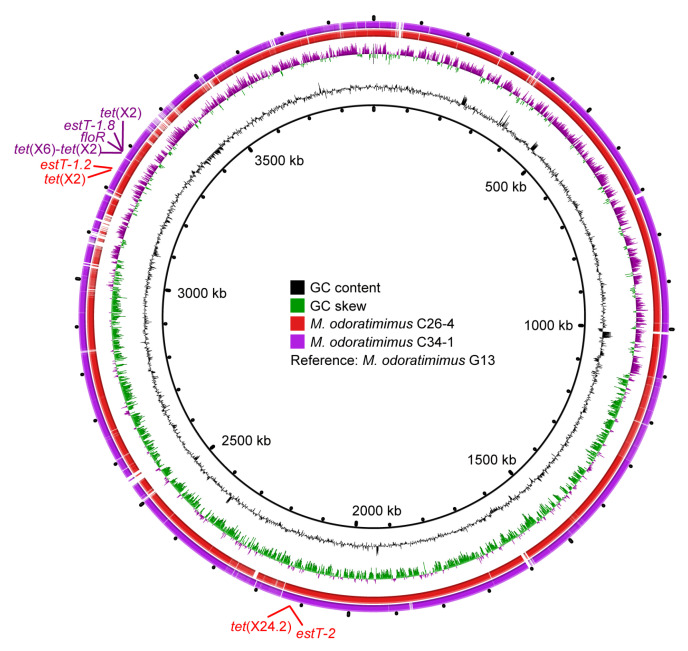
Comparative analysis of the *tet*(X)-carrying *Myroides* chromosomes. GC content, GC skew, *M. odoratimimus* C26-4 (CP182307), and *M. odoratimimus* C34-1 (CP182237) are from inside out, with *M. odoratimimus* G13 (CP037427) as the reference strain. Antibiotic resistance gene clusters for *M. odoratimimus* C26-4 and *M. odoratimimus* C34-1 are also marked in red and purple, respectively.

To analyze the molecular location of *tet*(X) and *estT* genes, NovaSeq and Nanopore sequencing of *M. odoratimimus* C26-4 and C34-1 were conducted. WGS results of *M. odoratimimus* C26-4 indicated the *tet*(X2) and *tet*(X24.2) genes were distantly located on a single chromosome (3,956,267 bp, CP182307), together with macrolide resistance genes *estT-1.2* and *estT-2*, respectively (Figure 3). In *M. odoratimimus* C34-1, the *tet*(X2) and *tet*(X6)-*tet*(X2) genes were tandemly located on a single chromosome (3,983,038 bp, CP182237), together with *estT-1.8* and phenicol resistance gene *floR* (Figure 3). A further comparative analysis confirmed they were highly homologous to the reference chromosome (3894807 bp, CP037427) of water-derived *M. odoratimimus* G13 (Figure 3), which also contained an untypable plasmid (43223 bp, CP037428).

**Figure 4:**
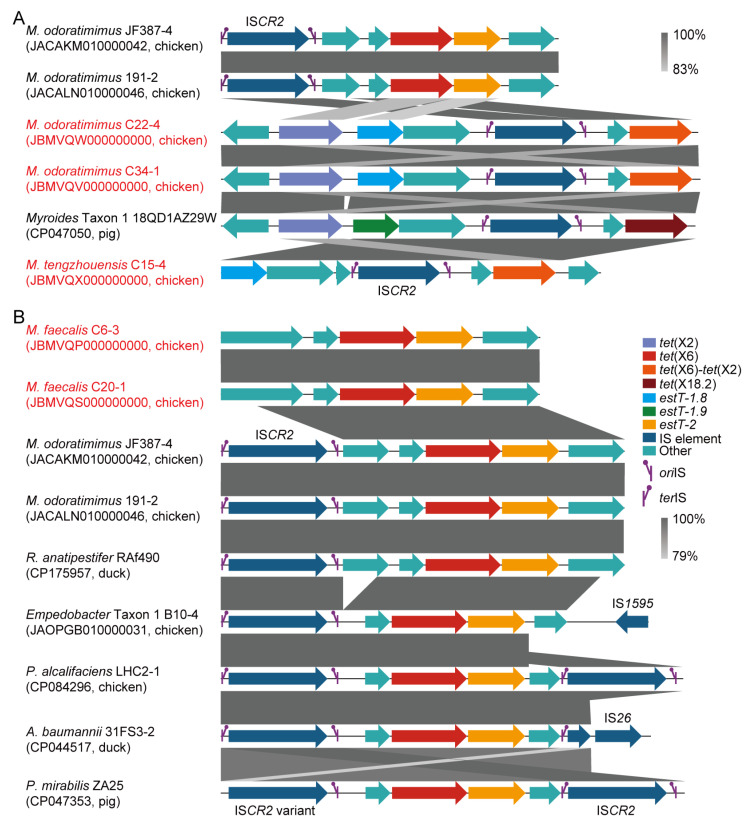
Genetic characteristics of *tet*(X) and *estT* genes. (**A**) IS*CR2*-associated *tet*(X) and *estT* loci in *Myroides* species, with regions sharing >83% nucleotide identity highlighted in grey. (**B**) Interspecific occurrence of *tet*(X6) and *estT-2*, with conserved regions sharing >79% nucleotide identity colored in grey. The strains highlighted in red are *Myroides* spp. we isolated in this study. The *tet*(X), *estT*, IS element, IS*CR2* replication initiation site (*ori*IS), IS*CR2* replication termination site (*ter*IS), and other genes are also indicated in different colors.

By analyzing the insertion sequence (IS), 6 out of 95 (6.3%) *tet*(X)-positive *Myroides* sp. strains were positive for IS*CR2*. In detail, the IS*CR2*-mediated transposon units of *tet*(X6)/*estT-2*, *tet*(X2)/*tet*(X6)-*tet*(X2)/*estT-1.8*, *tet*(X2)/*tet*(X18.2)/*estT-1.9*, and *tet*(X6)-*tet*(X2)/*estT-1.8* were identified in *M. odoratimimus* (e.g., JACAKM010000042), *M. tengzhouensis* (e.g., JBMVQX000000000), and *Myroides* Taxon 1 (e.g., CP047050; Figure 4A). Additionally, an IS*CR2*-mediated transposon of *tet*(X6)/*estT-2* was identified across *M. odoratimimus* (e.g., JACAKM010000042), *Riemerella anatipestifer* (e.g., CP175957), *Empedobacter* Taxon 1 (e.g., JAOPGB010000031), *Providencia alcalifaciens* (e.g., CP084296), *Acinetobacter baumannii* (e.g., CP044517), and *Proteus mirabilis* (e.g., CP047353) from food-producing animals in the NCBI database (Figure 4B). However, the *tet*(X) genes failed to be transferred into *A. baylyi* ADP1, *E. coli* C600, and *M. odoratimimus* ATCC BAA-634 by conjugation or natural transformation in this study. For the remaining 89 strains, all were negative for IS*CR2,* and their horizontal transmission risk also needed to be further confirmed.

## 4. Discussion

*Myroides* spp., especially *M. odoratimimus*, appear as a group of sporadically reported clinical pathogens [2,6]. Before this study, the *tet*(X) genes had been reported in *M. odoratimimus*, *M. odoratus*, and *M. phaeus* isolates from homo sapiens, animal, and environmental samples [6,13,22,23,51]. It is noted that our study demonstrated a low prevalence of *tet*(X)-positive MDR *M. odoratimimus*, *M. tengzhouensis*, *M. faecalis*, and *M. zaozhuangensis* in chicken samples. Worrisomely, the *tet*(X)-positive *M. odoratimimus*, *M. marinus*, *M. phaeus*, *M. pelagicus*, *M. injenensis*, *M. zaozhuangensis*, and *Myroides* Taxon 1–4 genomes were detected in the public NCBI database. These *tet*(X)-positive *Myroides* species highlighted the genomic diversity, which is seriously underestimated. According to the isolation source, animals accounted for the majority (77.9%) of 95 *tet*(X)-positive *Myroides* sp. strains, and an SNP-based phylogenetic tree indicated that clonal transmission rarely occurs, underlying the zoonotic driver of horizontal transmission of *tet*(X) genes in *Myroides* species.

Since the first report in *Bacteroides fragilis* [52], a total of 54 non-duplicate *tet*(X) variants have been detected in a variety of Gram-negative bacteria, indicating the rapid evolutionary rate over the past decades (Figure S3). Structurally, a combination of six key amino acid substitutions of Tet(X) proteins has been reported, leading to enhanced degradation activity [49,50]. In this study, we identified eight *tet*(X) variants in *Myroides* spp., of which *tet*(X6), *tet*(X6)-*tet*(X2), and *tet*(X24.2) were confirmed to confer tigecycline resistance, and *tet*(X18.2) with six similar amino acid substitutions was also speculated for tigecycline resistance. With the continuous emergence of *tet*(X) variants, their differences in resistance phenotypes need to be evaluated and focused [53,54].

For *estT* genes (Figure S4), there have been 15 non-duplicate variants since the first report in *Sphingobacterium faecium* from water in 2023 [55]. Here, we reported eight *estT* variants in *tet*(X)-positive *Myroides* sp. strains, including four novel variants, and *estT-1.2*, *estT-1.8*, and *estT-2* exhibited 2–4-fold increases of MICs against 16-atom-containing macrolides. In contrast, the *estT* genes have a similar three-dimensional structure and antibacterial activity to those of previously reported *tet*(X)-positive *Empedobacter* spp., but their hydrolyzation mechanisms need to be confirmed [18]. All the available data indicated that *Myroides* species act as the reservoir of *tet*(X) and *estT* gene clusters. There is no evidence for their co-expression or co-regulation; however, the co-existence of functional *tet*(X) and *estT* genes in this study signifies the co-dissemination risk.

IS*CR2* is an atypical insertion sequence that can transpose adjacent ARGs [e.g., *floR* and *sul2*] through a rolling-circle transposition process [56]. Despite the low frequency of *Myroides* species, 99% and 100% of *tet*(X) genes were mediated by IS*CR2* in *Acinetobacter* species and *E. coli*, respectively [45,57]. Our previous studies confirmed the IS*CR2*-mediated transposition ability of *tet*(X3), *tet*(X4), and *tet*(X5) genes in *Acinetobacter* species and *Aeromonas caviae* [12,45,58]. Although failing to be transferred by conjugation in this study, we identified a series of IS*CR2*-mediated transposon units of *tet*(X) and *estT* variants in *M. odoratimimus*, *M. tengzhouensis*, and *Myroides* Taxon 1. Additionally, a similar IS*CR2*-mediated transposition structure of *tet*(X6) and *estT-2* was detected in public *Myroides*, *Riemerella*, *Empedobacter*, *Providencia*, *Acinetobacter*, *and Proteus* genomes, indicating a risk of interspecies dissemination [12,16,18]. With the widespread use and residue in animals, humans, and environments, tetracycline and macrolide antibiotics may facilitate the formation and mobilization of *tet*(X)/*estT* gene clusters, but further exploration is needed.

## 5. Conclusions

In summary, this study highlights *Myroides* spp. as critical reservoirs of diverse *tet*(X) and *estT* variants, driving resistance to tetracyclines and macrolides, especially in poultry settings. The identification of novel *tet*(X), *estT*, and *Myroides* species, alongside IS*CR2*-mediated transposons, underscores the genomic plasticity enabling resistance dissemination across species boundaries. Despite non-conjugative *tet*(X) genes, their integration with IS*CR2* poses a significant risk for horizontal gene transfer. Our findings advocate stricter antimicrobial stewardship and genomic monitoring to mitigate the global spread of MDR *Myroides* sp. pathogens, reinforcing the One Health framework in addressing antibiotic resistance crises.

## Figures and Tables

**Table 1:** MICs of *tet*(X)-positive *Myroides* sp. isolates.

Antibiotics	Strains (MICs, mg/L) ^1^								
	C8-3	C6-3	C34-1	C26-4	C22-4	C21-3	C18-2	C20-1	C15-4
TC	32	128	64	128	64	64	64	64	32
TGC	4	8	16	16	16	16	16	8	8
SAM	2/1	2/1	32/16	16/8	32/16	16/8	32/16	2/1	16/8
CAZ	32	32	64	128	64	128	128	32	64
MEM	0.25	0.5	0.5	4	2	2	2	0.5	4
CTX	16	16	128	128	128	128	128	16	64
AMK	>256	64	256	>256	>256	>256	>256	256	>256
GEN	128	64	>256	128	>256	>256	256	128	256
CS	>256	256	>256	>256	>256	>256	>256	256	256
CIP	32	64	>64	>64	>64	>64	>64	>64	8
GAT	8	>16	>16	8	>16	8	8	>16	8
FFC	32	32	128	64	32	32	64	32	32
SXT	16/304	8/152	16/304	>16/304	16/304	>16/304	16/304	8/152	16/304
TYL	512	128	256	512	256	512	512	256	512
TIL	64	32	1	64	8	64	64	32	1
TIP	32	32	8	16	8	8	32	32	4

^1^ TC, tetracycline; TGC, tigecycline; SAM, ampicillin-sulbactam; CAZ, ceftazidime; MEM, meropenem; CTX, cefotaxime; AMK, amikacin; GEN, gentamicin; CS, colistin; CIP, ciprofloxacin; GAT, gatifloxacin; FFC, florfenicol; SXT, trimethoprim-sulfamethoxazole; TYL, tylosin; TIL, tilmicosin; TIP, tildipirosin.

**Table 2:** MICs of the *tet*(X) and *estT* clones.

Clones	MICs (mg/L) ^1^						
	TC	DOX	MIN	TGC	TYL	TIL	TIP
*E. coli* JM109 + pBAD24: *tet*(X2)	16	4	0.5	0.25	-	-	-
*E. coli* JM109 + pBAD24: *tet*(X6)	128	16	16	8	-	-	-
*E. coli* JM109 + pBAD24: *tet*(X6)-*tet*(X2)	128	16	16	4	-	-	-
*E. coli* JM109 + pBAD24: *tet*(X24.2)	128	32	16	8	-	-	-
*E. coli* JM109 + pBAD24: *estT-1.2*	-	-	-	-	512	64	1
*E. coli* JM109 + pBAD24: *estT-1.8*	-	-	-	-	512	64	2
*E. coli* JM109 + pBAD24: *estT-2*	-	-	-	-	256	64	1
*E. coli* JM109 + pBAD24	2	0.5	0.25	0.125	128	16	0.5

^1^ TC, tetracycline; DOX, doxycycline; MIN, minocycline; TGC, tigecycline; TYL, tylosin; TIL, tilmicosin; TIP, tildipirosin.

## Data Availability

Whole-genome sequences of nine *tet*(X)-positive *Myroides* sp. isolates are available in the NCBI BioProject repository (PRJNA1224301). Circular chromosome sequences of *M. odoratimimus* strains C26-4 (PRJNA1224307) and C34-1 (PRJNA1224310) are also submitted.

## Supplementary Materials

The following supporting information can be downloaded at: https://www.mdpi.com/article/10.3390/microorganisms13061180/s1, Figure S1: Three-dimensional structure of Tet(X) proteins; Figure S2: Three-dimensional structure of EstT proteins; Figure S3: Phylogenetic tree of the *tet*(X) variants; Figure S4: Phylogenetic tree of the *estT* variants; Table S1: Primers designed in this study; Table S2: Phenotypic characteristics of the novel *Myroides* species.

## Abbreviations

The following abbreviations are used in this manuscript:

MDR	Multidrug-resistant
MICs	Minimum inhibitory concentrations
LB	Luria–Bertani
MH	Mueller–Hinton
WGS	Whole genome sequencing
ANI	Average nucleotide identity
SNP	Single-nucleotide polymorphism
*is*DDH	in silico DNA-DNA hybridization
ARGs	Antibiotic resistance genes
ERIC-PCR	Enterobacterial repetitive intergenic consensus PCR
IS	Insertion sequence

## References

[1] LPSN. https://lpsn.dsmz.de/genus/myroides.

[2] Gunzer F., Rudolph W.W., Bunk B., Schober I., Peters S., Muller T., Oberheitmann B., Schrottner P. (2018). Whole-genome sequencing of a large collection of *Myroides odoratimimus* and *Myroides odoratus* isolates and antimicrobial susceptibility studies. Emerg. Microbes Infect..

[3] Aworh M.K., Colín-Castro C.A., Ortiz-Álvarez J.M., Hernández-Pérez C.F., Hernández-Durán M., García-Hernández M.d.L., Martínez-Zavaleta M.G., Becerra-Lobato N., Cervantes-Hernández M.I., Rosas-Alquicira G. (2024). *Myroides* species, pathogenic spectrum and clinical microbiology sight in Mexican isolates. PLoS ONE.

[4] Kurt A.F., Mete B., Houssein F.M., Tok Y., Kuskucu M.A., Yucebag E., Urkmez S., Tabak F., Aygun G. (2022). A pan-resistant *Myroides odoratimimus* catheter-related bacteremia in a COVID-19 patient and review of the literature. Acta Microbiol. Immunol. Hung..

[5] Sahu C., Patel S.S., Chaudhary R., Bhartiya C., Bhatnagar N. (2024). A Retrospective Study on UTI by *Myroides* Species: An Emerging Drug Resistant Nosocomial Pathogen. Indian J. Crit. Care M.

[6] Zhang P., Liu M., Fu J., Zhong C., Zong G., Cao G. (2020). Identification of a mobilizable, multidrug-resistant genomic island in *Myroides odoratimimus* isolated from Tibetan pasture. Sci. Total Environ..

[7] Aygar I.S., Aydogan C.N., Ozcan H., Unat I., Fatsa T., Tekin K., Yalci A., Hosbul T., Sahiner F., Gumral R. (2023). *Myroides odoratimimus*: A New Threat with Persistent Infections, Multidrug Resistance, and the Potential for Hospital Outbreaks. Jpn. J. Infect. Dis..

[8] Yartasi E., Durmaz R., Ari O., Mumcuoglu I., Dinc B. (2023). Molecular characterization of the multi-drug resistant *Myroides odoratimimus* isolates: A whole genome sequence-based study to confirm carbapenem resistance. Int. Microbiol..

[9] Seifert H., Blondeau J., Lucassen K., Utt E.A. (2022). Global update on the in vitro activity of tigecycline and comparators against isolates of *Acinetobacter baumannii* and rates of resistant phenotypes (2016–2018). J. Glob. Antimicrob. Res..

[10] Nguyen F., Starosta A.L., Arenz S., Sohmen D., Donhofer A., Wilson D.N. (2014). Tetracycline antibiotics and resistance mechanisms. Biol. Chem..

[11] Pfaller M.A., Huband M.D., Streit J.M., Flamm R.K., Sader H.S. (2018). Surveillance of tigecycline activity tested against clinical isolates from a global (North America, Europe, Latin America and Asia-Pacific) collection (2016). Int. J. Antimicrob. Agents.

[12] Chen C., Cui C.Y., Wu X.T., Fang L.X., He Q., He B., Long T.F., Liao X.P., Chen L., Liu Y.H. (2021). Spread of *tet*(X5) and *tet*(X6) genes in multidrug-resistant *Acinetobacter baumannii* strains of animal origin. Vet. Microbiol..

[13] He T., Wang R., Liu D.J., Walsh T.R., Zhang R., Lv Y., Ke Y.B., Ji Q.J., Wei R.C., Liu Z.H. (2019). Emergence of plasmid-mediated high-level tigecycline resistance genes in animals and humans. Nat. Microbiol..

[14] Sun J., Chen C., Cui C.Y., Zhang Y., Liu X., Cui Z.H., Ma X.Y., Feng Y., Fang L.X., Lian X.L. (2019). Plasmid-encoded *tet*(X) genes that confer high-level tigecycline resistance in *Escherichia coli*. Nat. Microbiol..

[15] Wang L., Liu D., Lv Y., Cui L., Li Y., Li T., Song H., Hao Y., Shen J., Wang Y. (2019). Novel Plasmid-Mediated *tet*(X5) Gene Conferring Resistance to Tigecycline, Eravacycline, and Omadacycline in a Clinical *Acinetobacter baumannii* Isolate. Antimicrob. Agents Chemother..

[16] He D., Wang L., Zhao S., Liu L., Liu J., Hu G., Pan Y. (2020). A novel tigecycline resistance gene, *tet*(X6), on an SXT/R391 integrative and conjugative element in a *Proteus* genomospecies 6 isolate of retail meat origin. J. Antimicrob. Chemother..

[17] Li R., Peng K., Xiao X., Wang Y., Wang Z. (2021). Characterization of novel IS*Aba1*-bounded *tet*(X15)-bearing composite transposon Tn*6866* in *Acinetobacter variabilis*. J. Antimicrob. Chemother..

[18] Chen C., Lv Y., Wu T., Liu J., Guo Y., Huang J. (2024). Concurrence of Inactivation Enzyme-Encoding Genes *tet*(X), *bla*_EBR_, and *estT* in *Empedobacter* Species from Chickens and Surrounding Environments. Foods.

[19] Li R., Jiang Y., Peng K., Wang Y., Wang M., Liu Y., Wang Z. (2022). Phenotypic and genomic analysis reveals *Riemerella anatipestifer* as the potential reservoir of *tet*(X) variants. J. Antimicrob. Chemother..

[20] Jin H., Jia Q., Jin X., Zhu X., Wang M.-G., Sun R.-Y., Cui C. (2024). Identification of novel Tet(X6)-Tet(X2) recombinant variant in *Elizabethkingia meningoseptica* from a bullfrog farm and downstream river in China. Front. Microbiol..

[21] Lu X., Zhang L., Peng K., Wang Q., Liu R., Wang Z., Li R. (2023). Characterisation of a Novel Tigecycline Resistance Gene *tet*(X22) and its Coexistence with *bla*_NDM-1_ in a *Pseudomonas caeni* Isolate. Int. J. Antimicrob. Agents.

[22] Liu D., Zhai W., Song H., Fu Y., Schwarz S., He T., Bai L., Wang Y., Walsh T.R., Shen J. (2020). Identification of the novel tigecycline resistance gene *tet*(X6) and its variants in *Myroides*, *Acinetobacter* and *Proteus* of food animal origin. J. Antimicrob. Chemother..

[23] Dong N., Zeng Y., Cai C., Sun C., Lu J., Liu C., Zhou H., Sun Q., Shu L., Wang H. (2022). Prevalence, transmission, and molecular epidemiology of *tet*(X)-positive bacteria among humans, animals, and environmental niches in China: An epidemiological, and genomic-based study. Sci. Total Environ..

[24] Hsieh Y.C., Wu J.W., Chen Y.Y., Quyen T.L.T., Liao W.C., Li S.W., Chen Y.C., Pan Y.J. (2021). An Outbreak of *tet*(X6)-Carrying Tigecycline-Resistant *Acinetobacter baumannii* Isolates with a New Capsular Type at a Hospital in Taiwan. Antibiotics.

[25] Miyoshi T., Iwatsuki T., Naganuma T. (2005). Phylogenetic characterization of 16S rRNA gene clones from deep-groundwater microorganisms that pass through 0.2-micrometer-pore-size filters. Appl. Environ. Microb..

[26] CLSI (2022). CLSI Performance Standards for Antimicrobial Susceptibility Testing Guideline M100-Ed32.

[27] Al Atrouni A., Joly-Guillou M.L., Hamze M., Kempf M. (2016). Reservoirs of Non-*baumannii Acinetobacter* Species. Front. Microbiol..

[28] Bankevich A., Nurk S., Antipov D., Gurevich A.A., Dvorkin M., Kulikov A.S., Lesin V.M., Nikolenko S.I., Pham S., Prjibelski A.D. (2012). SPAdes: A new genome assembly algorithm and its applications to single-cell sequencing. J. Comput. Biol..

[29] Wick R.R., Judd L.M., Gorrie C.L., Holt K.E. (2017). Unicycler: Resolving bacterial genome assemblies from short and long sequencing reads. PLoS Comput. Biol..

[30] NCBI. https://www.ncbi.nlm.nih.gov/datasets/genome/.

[31] Parks D.H., Imelfort M., Skennerton C.T., Hugenholtz P., Tyson G.W. (2015). CheckM: Assessing the quality of microbial genomes recovered from isolates, single cells, and metagenomes. Genome Res..

[32] Gurevich A., Saveliev V., Vyahhi N., Tesler G. (2013). QUAST: Quality assessment tool for genome assemblies. Bioinformatics.

[33] Liu D., Zhang Y., Fan G., Sun D., Zhang X., Yu Z., Wang J., Wu L., Shi W., Ma J. (2022). IPGA: A handy integrated prokaryotes genome and pan-genome analysis web service. iMeta.

[34] Jain C., Rodriguez-R L.M., Phillippy A.M., Konstantinidis K.T., Aluru S. (2018). High throughput ANI analysis of 90K prokaryotic genomes reveals clear species boundaries. Nat. Commun..

[35] Meier-Kolthoff J.P., Carbasse J.S., Peinado-Olarte R.L., Göker M. (2022). TYGS and LPSN: A database tandem for fast and reliable genome-based classification and nomenclature of prokaryotes. Nucleic Acids Res..

[36] Seemann T. ABRicate: Mass Screening of Contigs for Antimicrobial Resistance or Virulence Genes. https://github.com/tseemann/abricate.

[37] Xu S., Dai Z., Guo P., Fu X., Liu S., Zhou L., Tang W., Feng T., Chen M., Zhan L. (2021). ggtreeExtra: Compact Visualization of Richly Annotated Phylogenetic Data. Mol. Biol. Evol..

[38] Hall R.M., Schwarz S. (2016). Resistance gene naming and numbering: Is it a new gene or not?. J. Antimicrob. Chemother..

[39] Tamura K., Peterson D., Peterson N., Stecher G., Nei M., Kumar S. (2011). MEGA5: Molecular evolutionary genetics analysis using maximum likelihood, evolutionary distance, and maximum parsimony methods. Mol. Biol. Evol..

[40] Waterhouse A., Bertoni M., Bienert S., Studer G., Tauriello G., Gumienny R., Heer F.T., de Beer T.A.P., Rempfer C., Bordoli L. (2018). SWISS-MODEL: Homology modelling of protein structures and complexes. Nucleic Acids Res..

[41] Aziz R.K., Bartels D., Best A.A., DeJongh M., Disz T., Edwards R.A., Formsma K., Gerdes S., Glass E.M., Kubal M. (2008). The RAST Server: Rapid annotations using subsystems technology. BMC Genom..

[42] Alikhan N.F., Petty N.K., Ben Zakour N.L., Beatson S.A. (2011). BLAST Ring Image Generator (BRIG): Simple prokaryote genome comparisons. BMC Genom..

[43] Sullivan M.J., Petty N.K., Beatson S.A. (2011). Easyfig: A genome comparison visualizer. Bioinformatics.

[44] Ram H., Kumar A., Thomas L., Dastager S.G., Mawlankar R., Singh V.P. (2015). *Myroides indicus* sp. nov., isolated from garden soil. Int. J. Syst. Evol. Microbiol..

[45] Chen C., Cui C.Y., Yu J.J., He Q., Wu X.T., He Y.Z., Cui Z.H., Li C., Jia Q.L., Shen X.G. (2020). Genetic diversity and characteristics of high-level tigecycline resistance Tet(X) in *Acinetobacter* species. Genome Med..

[46] Versalovic J., Koeuth T., Lupski R. (1991). Distribution of repetitive DNA sequences in eubacteria and application to finerpriting of bacterial genomes. Nucleic Acids Res..

[47] Grundmann H.J., Towner K.J., Dijkshoorn L., Gernersmidt P., Maher M., Seifert H., Vaneechoutte M. (1997). Multicenter Study Using Standardized Protocols and Reagents for Evaluation of Reproducibility of PCR-Based Fingerprinting of *Acinetobacter* spp.. J. Clin. Microbiol..

[48] Cui C.Y., Chen C., Liu B.T., He Q., Wu X.T., Sun R.Y., Zhang Y., Cui Z.H., Guo W.Y., Jia Q.L. (2020). Co-occurrence of Plasmid-Mediated Tigecycline and Carbapenem Resistance in *Acinetobacter* spp. from Waterfowls and Their Neighboring Environment. Antimicrob. Agents Chemother..

[49] Cui C.Y., He Q., Jia Q.L., Li C., Chen C., Wu X.T., Zhang X.J., Lin Z.Y., Zheng Z.J., Liao X.P. (2021). Evolutionary Trajectory of the Tet(X) Family: Critical Residue Changes towards High-Level Tigecycline Resistance. mSystems.

[50] Cheng Q., Cheung Y., Liu C., Chan E.W.C., Wong K.Y., Zhang R., Chen S. (2022). Functional and phylogenetic analysis of TetX variants to design a new classification system. Commun. Biol..

[51] Ming D., Chen Q.Q., Chen X.T. (2017). Analysis of resistance genes in pan-resistant *Myroides odoratimimus* clinical strain PR63039 using whole genome sequencing. Microb. Pathog..

[52] Speer B.S., Bedzyk L., Salyers A.A. (1991). Evidence that a novel tetracycline resistance gene found on two *Bacteroides* transposons encodes an NADP-requiring oxidoreductase. J. Bacteriol..

[53] Linkevicius M., Sandegren L., Andersson D.I. (2016). Potential of Tetracycline Resistance Proteins To Evolve Tigecycline Resistance. Antimicrob. Agents Chemother..

[54] Blake K.S., Xue Y.-P., Gillespie V.J., Fishbein S.R.S., Tolia N.H., Wencewicz T.A., Dantas G. (2025). The tetracycline resistome is shaped by selection for specific resistance mechanisms by each antibiotic generation. Nat. Commun..

[55] Dhindwal P., Thompson C., Kos D., Planedin K., Jain R., Jelinski M., Ruzzini A. (2023). A neglected and emerging antimicrobial resistance gene encodes for a serine-dependent macrolide esterase. Proc. Natl. Acad. Sci. USA.

[56] Toleman M.A., Bennett P.M., Walsh T.R. (2006). IS*CR* elements: Novel gene-capturing systems of the 21st century?. Microbiol. Mol. Biol. R.

[57] Zhang Z., Zhan Z., Shi C. (2022). International Spread of Tet(X4)-Producing *Escherichia coli* Isolates. Foods.

[58] Chen C., Chen L., Zhang Y., Cui C.Y., Wu X.T., He Q., Liao X.P., Liu Y.H., Sun J. (2019). Detection of chromosome-mediated *tet*(X4)-carrying *Aeromonas caviae* in a sewage sample from a chicken farm. J. Antimicrob. Chemother..

