# Supplementary material for: Genomic Diversity of the tet(X)-Positive Myroides Species
Source: Microorganisms. 2025 May 22;13(6):1180. doi: 10.3390/microorganisms13061180 (PMC12195491; doi:10.3390/microorganisms13061180)
Supplement: Supplementary file 1 [file microorganisms-13-01180-s001.zip › microorganisms-3593330-supplementary.pdf]

Supplementary information

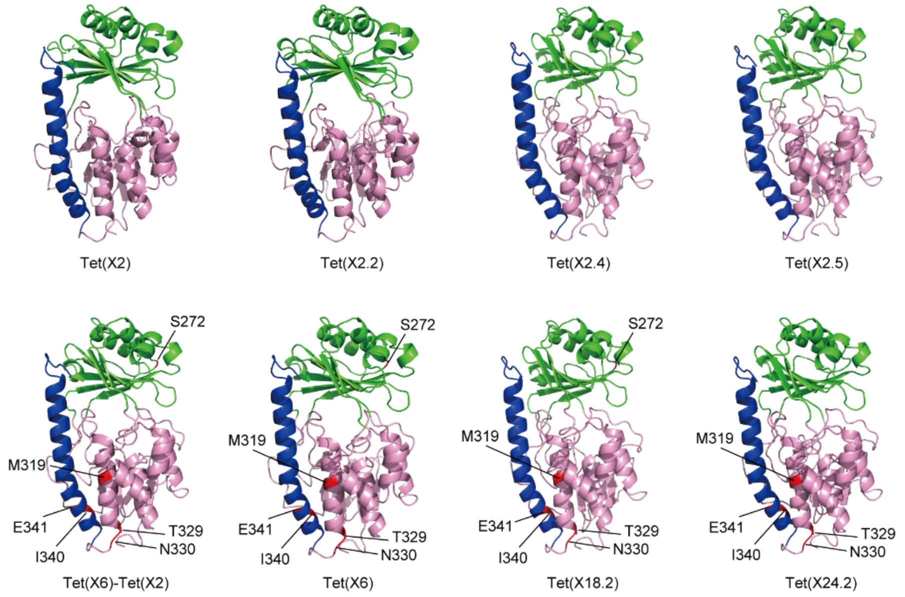

Figure S1. Three-dimensional structure of Tet(X) proteins. The substrate-binding domain, FAD-binding domain, and C-terminal helix are colored in green, pink, and blue, respectively, and the reported key amino acid sites are also marked.

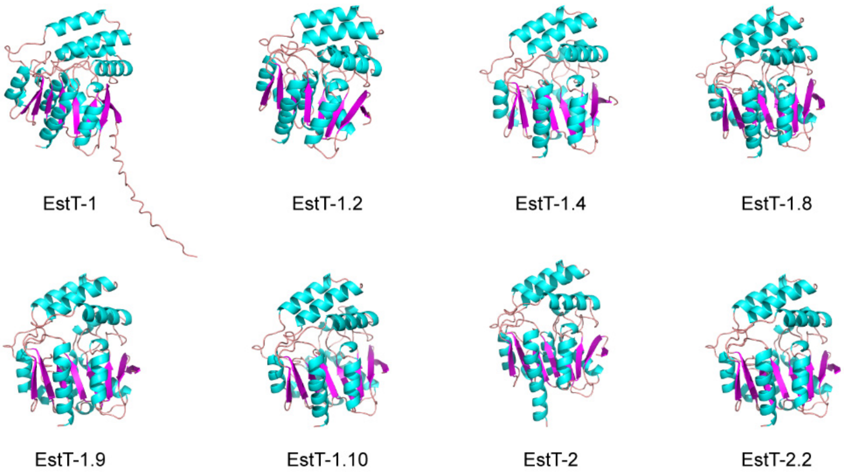

Figure S2. Three-dimensional structure of EstT proteins. The α-helix, β-sheet, and random coli are highlighted in cyan, purple, and pink, respectively.

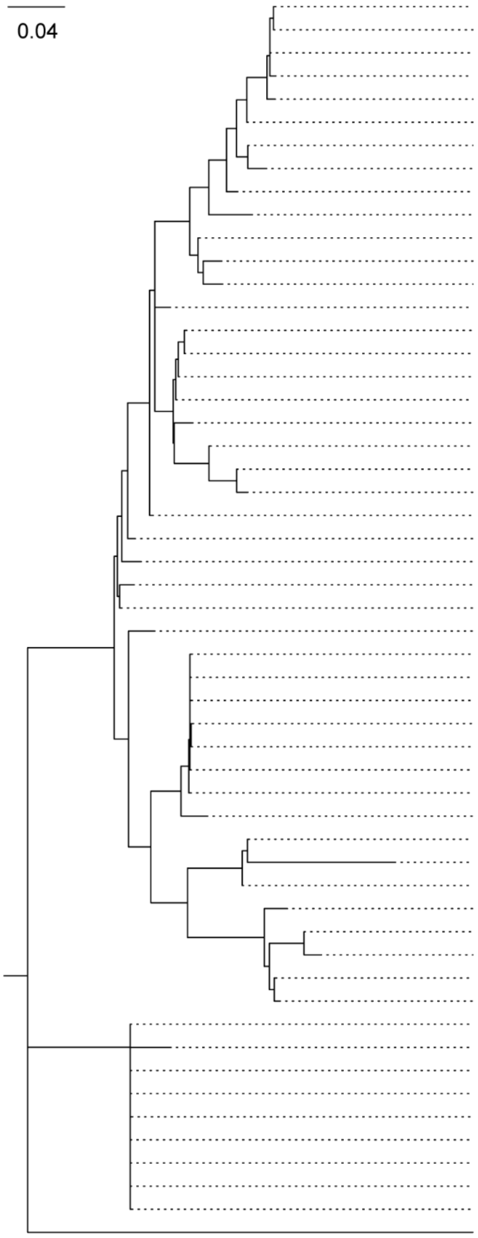

Variants	Accession numbers	Sizes
tet(X6.2)	CP081928	1137 bp
tet(X6.3)	CP081929	1137 bp
tet(X6)/*tet*(X5.2)	MN507533/CP048670	1137 bp
tet(X13)	KU547125	1137 bp
tet(X6.4)	AP025181	1137 bp
tet(X6.5)	CP085032	1137 bp
tet(X7)	KU547176	1137 bp
tet(X9)	MT292309	1137 bp
tet(X5.3)/*tet*(X6) variant	CP048661/CP048828	1137 bp
tet(X8)	KU548516	1137 bp
tet(X5.4)	CP094261	1167 bp
tet(X5)	CP040912	1167 bp
tet(X15)	CP060811	1158 bp
tet(X11)	MT292307	1167 bp
tet(X14.3)	CP081929	1167 bp
tet(X14.4)	JAGFUF010000016	1167 bp
tet(X14.2)	CP007503	1167 bp
tet(X14)	CP053698	1167 bp
tet(X23)	JAOPGT000000000	1137 bp
tet(X18)	CP072192	1137 bp
tet(X18.2)	CP047050	1137 bp
tet(X6)-*tet*(X2)	JBCFCM010000007	1137 bp
tet(X19)	CP081923	1167 bp
tet(X20)	JAGFUR010000001	1167 bp
tet(X4)	MK134376	1158 bp
tet(X12)	KU548377	1167 bp
tet(X12.2)	CP072185	1167 bp
tet(X16)	JAGFUM010000001	1167 bp
tet(X2.2)	JAOPGU000000000	1167 bp
tet(X2.3)	CP040909	1137 bp
tet(X2.5)	JACAGU010000000	1167 bp
tet(X0)	M37699	1167 bp
tet(X2.4)	JACAGS010000000	1167 bp
tet(X2)/*tet*(X10)	AJ311171/KU548536	1167 bp
tet(X10.2)	CP081927	1167 bp
tet(X21)	CP072188	1167 bp
tet(X17.2)	CP081928	1167 bp
tet(X22)	CP114976	1137 bp
tet(X17)	JAGFUH010000017	1167 bp
tet(X25)	JAOPGI000000000	1167 bp
tet(X24)	JAOPGW000000000	1167 bp
tet(X24.2)	CP182307	1137 bp
tet(X26)	CP106831	1167 bp
tet(X26.2)	JAOPGR000000000	1167 bp
tet(X3.4)	CP094246	1164 bp
tet(X3.2)	CP043635	1167 bp
tet(X3)	MK134375	1167 bp
tet(X3.3)	CP094246	1166 bp
tet(X3.7)	CP094838	1167 bp
tet(X3.5)	CP094246	1165 bp
tet(X3.6)	CP094246	1165 bp
tet(X3.8)	CP094838	1165 bp
tet(X3.9)	CP094838	1167 bp
tet(X1)	AJ311171	1080 bp

Figure S3. Phylogenetic tree of the *tet*(X) variants. GenBank accession numbers and nucleotide sequence lengths of 54 non-duplicate *tet*(X) variants are provided. Bar, 0.04 nucleotide substitutions per site.

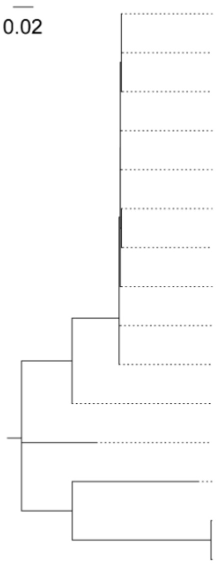

Variants	Accession numbers	Sizes
estT-1.6	JACXZC010000029	915 bp
estT-1.10	JACALG010000000	843 bp
estT-1.9	CP047050	843 bp
estT-1.3	JAOPGN000000000	915 bp
estT-1.8	JBMVQX000000000	843 bp
estT-1.4	JAOPGS000000000	843 bp
estT-1.7	CP104209	843 bp
estT-1.5	CP082272	849 bp
estT-1.2	JAOPGW000000000	849 bp
estT-1	CP094932	921 bp
estT-3	JAOPHF000000000	921 bp
estT-4	JAOPHD000000000	918 bp
estT-5	JAOPGR000000000	915 bp
estT-2	JAOPGC000000000	861 bp
estT-2.2	JACALF000000000	861 bp

Figure S4. Phylogenetic tree of the *estT* variants. GenBank accession numbers and nucleotide sequence lengths of 15 non-duplicate *estT* variants are provided. Bar, 0.02 nucleotide substitutions per site.

Table S1. Primers designed in this study.

Primers	Nucleotide sequences (5′ to 3′) [1]	Sizes
tet(X24.2)-F	ttgggctagcaggaggaattcATGAATGTATTAAACAATAAAAAAATCGC	1137 bp
tet(X24.2)-R	cttgcatgcctgcaggtcgacTTATAGATTCATTAGTTTTTGGAAAGAAAA	
estT-1.8-F	tacccgtttttttgggctagcATGAAAGAGAAAATAATTAAAACAAACGG	843 bp
estT-1.8-R	cttgcatgcctgcaggtcgacTCAGTCATTTATGTGTTTTTCTATTCCA	

[1] The homologous sequences are underlined.

Table S2. Phenotypic characteristics of the novel *Myroides* species.

**Species**	***Myroides tengzhouensis***	***Myroides faecalis***	***Myroides zaozhuangensis***	***Myroides odoratimimus***
Type strain	C15-4	C20-1	C8-3	ATCC BAA-634
Gram-staining	Red	Red	Red	Red
Morphology	Rod-shaped	Rod-shaped	Rod-shaped	Rod-shaped
Colony	Light yellow, circular, smooth surface, and regular margin	Light yellow, circular, smooth surface, and regular margin	Light yellow, circular, smooth surface, and regular margin	Light yellow, circular, smooth surface, and regular margin
O_2	+	+	+	+
pH	6-8	6-8	5-9	6-8
NaCl	≤3%	≤4%	≤4%	≤4%
Temperature	≤38 ℃	≤38 ℃	≤41 ℃	≤39 ℃
Motility	-	-	-	-
Hemolysis	-	-	-	-
Glucose	-	-	-	-
Oxidase	+	+	+	+
Citrate	-	-	-	-
Maltose	-	-	-	-
Arginine dihydrolase	+	+	+	+
Mannitol	-	-	-	-
Xylose	-	-	-	-
Nitrate reduction	-	-	-	-
DNA	+	+	+	+
Acetamide	+	+	+	+

+, positive; -, negative.
